# 

**Published:** 1919-12-27

**Authors:** 


### Monmouthshire Medical Appointments.

Miss Grace Leisk Hunter, M.B., Ch.B., of Saltcoats, Ayrshire, who has had experience at Bolton, Glasgow, and Paisley; Mr. Harold Ellis, M.R.C.S. (Eng.), L.R.C.P. (Lond.), M.B. (Lond.), assistant curator and assistant demonstrator in pathology, St. George's Hospital, London; and Mr. Purser Davies, M.B., Ch.B. (Edin.), of Chester, medical officer in charge of the prisoners of war camp and Royal Army Ordnance Depôt, Bromley, have been appointed assistant medical officers by the Monmouthshire Education Committee. Mr. Harold Wishaw Wallis, L.D.S., R.C.S. (Eng.), of Cardiff, was appointed school dentist.

### The College of Nursing
### (Limited by Guarantee).

LIVERPOOL CENTRE.

An interesting lecture on child welfare was delivered by Dr. Macalister to members of the Liverpool Centre, in the Lecture Theatre of the Royal Infirmary, Liverpool, on Wednesday, December 10.

At a members' meeting held previous to the lecture, Miss Cummins, R.R.C., in the chair, it was unanimously resolved that a letter of thanks be written to the Finance Committee expressing the appreciation of the members for the Club which had been provided through the Committee's kind and generous efforts. The Club is at 38 Church Street, and is open from 10 A.M. to 8 P.M. Any meal may be ordered and brought to members from the café below.

A cordial vote of thanks was passed to Miss Cummins, the Chairman, and Miss Worsley, the Hon. Secretary, for their services in connection with the Club, which has proved a great satisfaction to the members.

### Dinner to Dame Sidney Browne.

The wish having been expressed that the photograph of the dinner to Dame Sidney Browne, G.B.E., held on December 3, 1919, which was published in our issue of December 13, should be reproduced so as to be readily available, we have pleasure in announcing that the publishers of The Hospital have arranged for this to be done. The photograph, which is full page, is printed in sepia on a cream card, measuring 12½ by 10 inches, and will be sent post free, securely packed, for 1s. Orders should be addressed to the publishers of The Hospital, The Scientific Press, 28 & 29 Southampton Street, Strand, London, W.C.

It is a wonderfully reproduced picture, in which everyone present stands out clearly, and the whole constitutes one of the most attractive reproductions relating to the war.

### The Financial Position of Voluntary Hospitals.

We hear from authoritative sources that hospital funds have derived substantial benefit from the wide newspaper publicity recently given to their claims by the proprietors of Bovril.

